# Comparison of gene expression signatures of diamide, H_2_O_2 _and menadione exposed *Aspergillus nidulans *cultures – linking genome-wide transcriptional changes to cellular physiology

**DOI:** 10.1186/1471-2164-6-182

**Published:** 2005-12-20

**Authors:** István Pócsi, Márton Miskei, Zsolt Karányi, Tamás Emri, Patricia Ayoubi, Tünde Pusztahelyi, György Balla, Rolf A Prade

**Affiliations:** 1 Department of Microbiology and Biotechnology, Faculty of Science, University of Debrecen, P.O.Box 63, H-4010 Debrecen, Hungary; 2 Department of Medicine, Faculty of Medicine, University of Debrecen, P.O. Box 19, H-4012 Debrecen, Hungary; 3 Department of Biochemistry and Molecular Biology, Oklahoma State University, 348E Noble Research Center, Stillwater, OK 74078, USA; 4 Department of Neonatology, Faculty of Medicine, University of Debrecen, P.O.Box 37; H-4012 Debrecen, Hungary; 5 Department of Microbiology and Molecular Genetics, Oklahoma State University, 307 LSE, Stillwater, OK 74078, USA

## Abstract

**Background:**

In addition to their cytotoxic nature, reactive oxygen species (ROS) are also signal molecules in diverse cellular processes in eukaryotic organisms. Linking genome-wide transcriptional changes to cellular physiology in oxidative stress-exposed *Aspergillus nidulans *cultures provides the opportunity to estimate the sizes of peroxide (O_2_^2-^), superoxide (O_2_^•-^) and glutathione/glutathione disulphide (GSH/GSSG) redox imbalance responses.

**Results:**

Genome-wide transcriptional changes triggered by diamide, H_2_O_2 _and menadione in *A. nidulans *vegetative tissues were recorded using DNA microarrays containing 3533 unique PCR-amplified probes. Evaluation of LOESS-normalized data indicated that 2499 gene probes were affected by at least one stress-inducing agent. The stress induced by diamide and H_2_O_2 _were pulse-like, with recovery after 1 h exposure time while no recovery was observed with menadione. The distribution of stress-responsive gene probes among major physiological functional categories was approximately the same for each agent. The gene group sizes solely responsive to changes in intracellular O_2_^2-^, O_2_^•- ^concentrations or to GSH/GSSG redox imbalance were estimated at 7.7, 32.6 and 13.0 %, respectively. Gene groups responsive to diamide, H_2_O_2 _and menadione treatments and gene groups influenced by GSH/GSSG, O_2_^2- ^and O_2_^•- ^were only partly overlapping with distinct enrichment profiles within functional categories. Changes in the GSH/GSSG redox state influenced expression of genes coding for PBS2 like MAPK kinase homologue, PSK2 kinase homologue, AtfA transcription factor, and many elements of ubiquitin tagging, cell division cycle regulators, translation machinery proteins, defense and stress proteins, transport proteins as well as many enzymes of the primary and secondary metabolisms. Meanwhile, a separate set of genes encoding transport proteins, CpcA and JlbA amino acid starvation-responsive transcription factors, and some elements of sexual development and sporulation was ROS responsive.

**Conclusion:**

The existence of separate O_2_^2-^, O_2_^•- ^and GSH/GSSG responsive gene groups in a eukaryotic genome has been demonstrated. Oxidant-triggered, genome-wide transcriptional changes should be analyzed considering changes in oxidative stress-responsive physiological conditions and not correlating them directly to the chemistry and concentrations of the oxidative stress-inducing agent.

## Background

There is experimental evidence that formation of oxidants is regulated in cells by factors, affecting targets specifically [1-4]. Different oxidative stress-inducing agents (cumene hydroperoxide, diamide, H_2_O_2_, linoleic acid 13-hydroperoxide, menadione), which disturb intracellular oxidant concentrations specifically, influence segments of the genome differentially with almost no overlap in *Saccharomyces cerevisiae*, when a genome-wide set of deletion strains was tested [5]. For example, respiration was shown to be important in H_2_O_2 _defense while the NADPH-producing pentose phosphate pathway was menadione-responsive [5].

Oxidant levels are clearly influenced by the chemical structure of the agents used to trigger stress as well as the concentration and exposure time of the agents [6,7]. Overdosing with reactants decreases specificity of treatments by increasing the number of affected oxidants and diminishing survival rates [6,7].

The filamentous fungus *Aspergillus nidulans *with whole genome sequence availability and extensive EST collections is an excellent model to perform transcriptome analyses using EST-based DNA microarrays [8-10]. Physiological parameters indicative of oxidative stress can also be measured with high reproducibility in *A. nidulans *[11,12].

In this study, we assay physiologically well-defined oxidative stress-inducing systems to describe the global transcriptional changes observable in *A. nidulans *vegetative hyphae exposed to diamide, H_2_O_2 _and menadione. In addition, we demonstrate large gene groups responsive to changes in intracellular peroxide (O_2_^2-^) and superoxide (O_2_^•-^) levels or glutathione/glutathione disulfide (GSH/GSSG) redox imbalance.

## Results

### Optimization of oxidative stress inducing conditions

For diamide, H_2_O_2 _and menadione treatments, 1.8, 75 and 0.8 mM were selected, respectively, which were well below the "*dosis lethalis minima*" (DLM) determined in liquid *A. nidulans *cultures (Additional file 1:Supplement1 for physiological changes).

**Figure 1:**
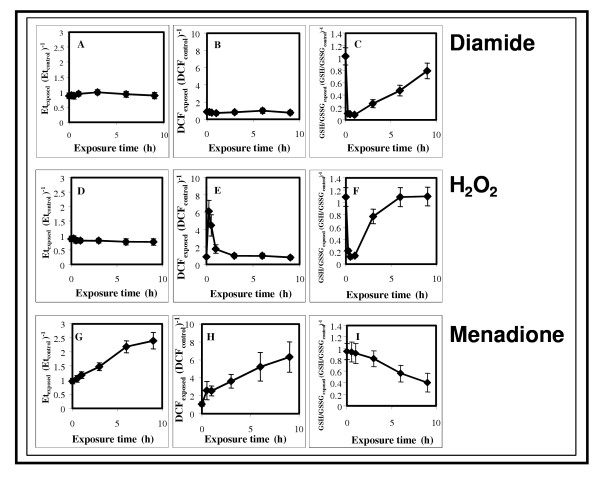
Time-course of physiological changes under diamide, H_2_O_2 _and menadione stress. Intracellular superoxide (**Parts A, D and G**), peroxide (**Parts B, E and H**) concentrations and GSH/GSSG values (**Parts C, F and I**) recorded under stress were factored with their counterparts found in untreated control cultures {Et_exposed _(Et_control_)^-1^; DCF_exposed _(DCF_control_^-1^); GSH/GSSG_exposed _(GSH/GSSG_control_)^-1^}. Intracellular superoxide and peroxide levels were characterized by specific Et (ethidium) and DCF (dichlorofluorescein) productions, respectively, in both stress-exposed and control cultures (Additional file 1:Supplement1 for physiological changes).

Diamide at 1.8 mM decreased GSH/GSSG value without effecting intracellular O_2_^2- ^or O_2_^•- ^levels (Figure 1, Additional file 1:Supplement1 for physiological changes). H_2_O_2 _and menadione increased intracellular O_2_^2- ^and O_2_^•-^levels, respectively, but also disturbed GSH/GSSG redox balance at all concentrations tested. In addition, menadione added at all tested concentrations facilitated intracellular accumulation of O_2_^2- ^(Figure 1, Additional file 1:Supplement1 for physiological changes). Increases in intracellular O_2_^2- ^levels induced by 75 mM H_2_O_2 _or 0.8 mM menadione and decreases in GSH/GSSG ratios when diamide, H_2_O_2 _or menadione was present were comparable to each other, respectively (Figure 1). The stress observed in diamide and H_2_O_2_-exposed cultures was pulse-like with a maximum intensity under 1 h exposure time and followed with full recovery (3–9 h). Meanwhile, all stress-related physiological parameters tested changed steadily up to 9 h exposure time in menadione-treated cultures indicating accumulation of stress with no recovery.

Short time (1 h) exposures to 1.8 mM diamide increased the specific glutathione *S*-transferase (GST) and catalase activities while superoxide dismutase (SOD) activity went up only after extended treatments (6 h; Additional file 1:Supplement1 for physiological changes). The specific SOD, GST and catalase activities all responded to short 0.8 mM menadione treatments (1 h) while 75 mM H_2_O_2 _triggered elevations only in catalase activity (Additional file 1:Supplement1 for physiological changes). After extended (6 h) incubation with H_2_O_2_, the increase in SOD and GST activities was significant at *P *< 5 % and *P *< 1 %, respectively (Additional file 1:Supplement1 for physiological changes).

Similar to previous observations [6,7], 1.8 mM diamide, 75 mM H_2_O_2 _and 0.8 mM menadione did not influence cell survival rates significantly even after extended (9 h) treatment periods (data not shown).

**Figure 2:**
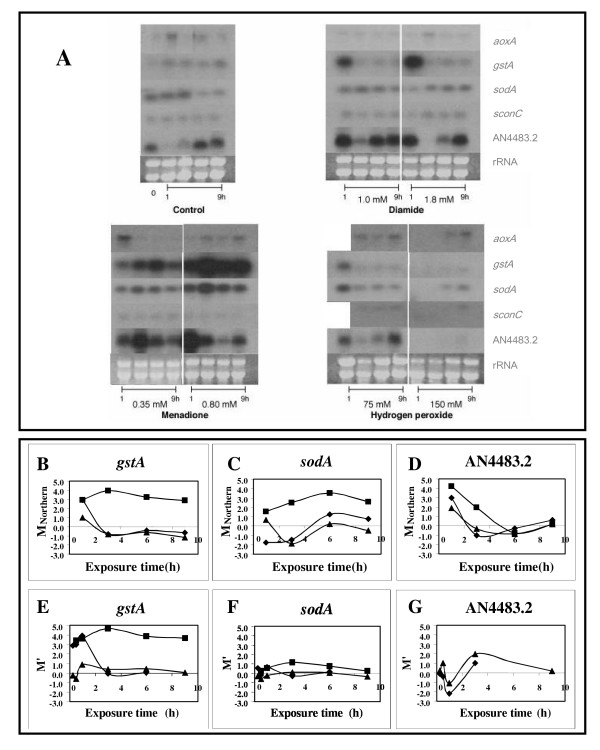
Dose-dependence and time-course of transcriptional effects of diamide, H_2_O_2 _and menadione on selected genes. **Part A. **Changes in the transcription of *aoxA*, *gstA*, *sodA*, *sconC *and a homologue of the Ca^2+^-calmodulin-dependent serine-threonine-protein kinase of *S. pombe *(ORF ID: AN4483.2) shown by Northern blot hybridization. **Parts B-D. **Graphical representation of optical density ratios of autoradiographic bands visualized on Northern blots in parallel stress-exposure and control experiments for *gstA*, *sodA *and the *A. nidulans *homologue of *S. pombe *Ca^2+^-calmodulin-dependent serine-threonine-protein kinase, respectively. By definition, M_Northern _= log_2_(optical density_stress-exposed_*optical density_control_^-1^). **Parts E-G. **Time-courses of the transcriptional changes recorded in DNA microarray experiments for *gstA *(ORF ID: AN4905.2, OSU contig ID: contig2000Sep131300_1307), *sodA *(ORF ID: AN0241.2, OSU contig ID: contig2000Sep131300_575) and the *A. nidulans *homologue of *S. pombe *Ca^2+^-calmodulin-dependent serine-threonine-protein kinase (ORF ID: AN4483.2, OSU contig ID: contig2000Sep131300_1404), respectively. Symbols ◆, ▲ and ■ stand for 1.8 mM diamide, 75 mM H_2_O_2 _and 0.8 mM menadione treatments, respectively.

Northern blot mRNA accumulation analysis of selected genes expressed under various kinds of oxidative stress are shown in Figure 2. M_Northern _= log_2_(optical density_stress-exposed_*optical density_control_^-1^) values were calculated for each exposure time and each stress-inducing agent concentration tested. Among the selected genes were: *gstA *(glutathione *S*-transferase) clearly up-regulated by diamide and menadione (Figures 2A and 2B); *sodA *(Cu,Zn-superoxide dismutase), which responded solely to menadione treatments (Figure 2A). In addition, *sodA *was down-regulated in control cultures after 6 h, which resulted in fluctuations in M_Northern _= f(t) functions when mycelia were exposed to diamide or H_2_O_2 _(Figures 2A and 2C). Expression of a Ca^2+^-calmodulin-dependent serine-threonine-protein kinase homologue of *Schizosaccharomyces pombe *(ORF ID: AN4483.2) fluctuated in both stress-exposed and control cultures annulling each other when M_Northern _= f(t) were calculated after 3 h (Figures 2A and 2D). Transcription of *sconC *(a sulfur metabolic transcriptional regulator) and *aoxA *(mitochondrial alternative oxidase) was not effected by oxidative stress with M_Northern _values around or between +1 and -1 (data not shown).

Gene activation or repression was dose-dependent when diamide and menadione concentrations were elevated from 1.0 to 1.8 mM and from 0.35 to 0.8 mM, respectively (Figure 2). When the concentration of H_2_O_2 _was elevated from 75 to 150 mM the mRNA pools were degraded at 1 and 3 h exposure times while there was apparently no reduction in rRNA levels at any exposure time tested (Figure 2).

### Evaluation of cDNA microarray gene expression data

**Figure 3:**
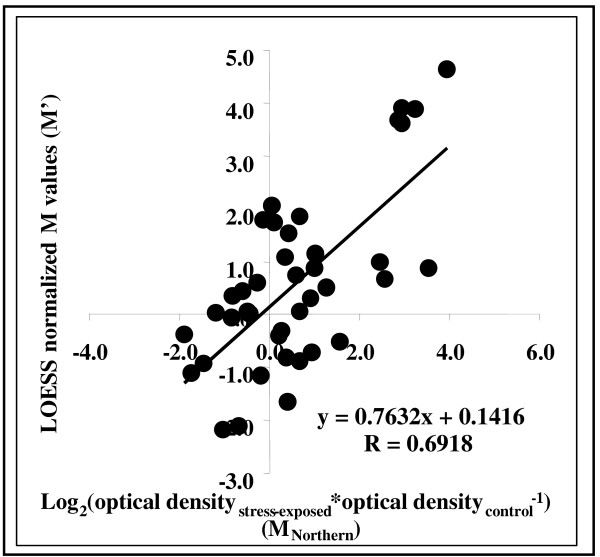
Correlation between the transcriptional changes recorded for selected genes (*aoxA*, *gstA*, *sodA *and *sconC*) in parallel DNA microarray and Northern blot hybridizations. In DNA microarray experiments, gene expressions were read on spots representing the following contigs: *aoxA *(ORF ID: AN2099.2): OSU contig ID: contig2000Sep131300_2956); *gstA *(ORF ID: AN4905.2): OSU contig ID: contig2000Sep131300_1307; *sodA *(ORF ID: AN0241.2): OSU contig ID: contig2000Sep131300_575, *sconC *(ORF ID: AN2302.2): contig2000Sep131300_757. All M'-M_Northern _data pairs are from 1.8 mM diamide, 75 mM H_2_O_2 _and 0.8 mM menadione treatments (1–9 h).

The log_2_-ratios of primary DNA microarray readings for the gene probes (M values) were normalized by LOESS intensity-dependent block-by-block normalization (M' values). The validity of the normalized DNA microarray readings was estimated by correlating DNA microarray M' and the appropriate M_Northern _values calculated for Northern blots (Figures 2 and 3). The correlation between gene expression data determined for the same set of randomly selected genes was R≈0.7 (Figure 3).

Time-dependence of gene expression profiles from cDNA microarrays showed that *gstA *was up-regulated by menadione and diamide while *sodA *was only induced by menadione (Figures 2E and 2F). The transcription of *sodA *was not fluctuating in the presence of H_2_O_2 _and diamide, and all M' values were within [+1,-1] thresholds (Figure 2F). The expression of Ca^2+^-calmodulin-dependent serine-threonine-protein kinase was clearly fluctuating in microarray experiments with a maximum down-regulation at 1 h incubation and with a maximum induction of 3 h incubation under diamide and H_2_O_2_-treatments (Figure 2G).

**Figure 4:**
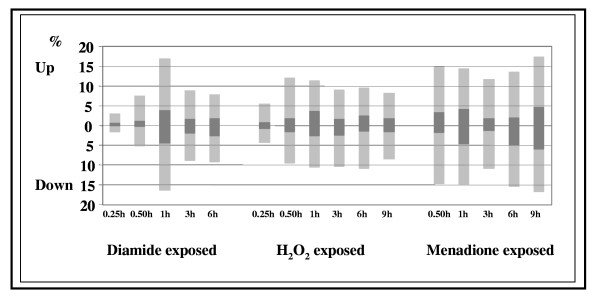
Ratios of gene probes with at least more than two-fold induction or repression. Global gene expression changes were recorded on DNA microarrays and are presented here as a function of oxidative stress-inducing agents and exposure times. Dark and light gray colors indicate the percentage of affected gene probes obeying either [+2;-2] (four-fold transcriptional change) or [+1;-1] (two-fold change) M' thresholds criteria, respectively.

Time-course of gene expression ratios, which was affected by stress-inducing agents more than two-fold, showed a maximum intensity response by 1 h exposure time (Figure 4). The expression ratio decreased sharply after 1 h exposure to diamide, decreased slightly and more slowly with H_2_O_2 _and remained high up to 9 h in the presence of menadione with a transient decrease at 3–6 h and a second peak at 9 h (Figure 4). With diamide and menadione, maximum 16.9–17.4 % of the gene probes responded with at least two-fold change in expression to stress, while this ratio was lower (maximum 12 %) for H_2_O_2_. It is noteworthy that 4–6 % of the M' values were below or above the [+2,-2] thresholds, *i.e*. higher than 4-fold change recorded, when the ratio of affected gene probes was maximal for the agents (Figure 4).

**Figure 5:**
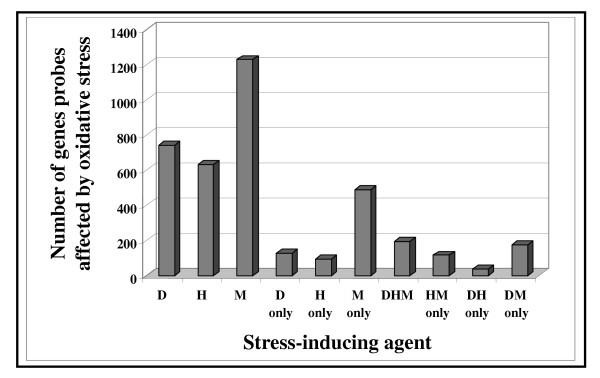
Distribution of selected oxidative stress-affected gene probes (1502 in total) as a function of stress-inducing agents. Gene probes were filtered using [+1;-1] M' thresholds criteria for transcriptional changes, and only the first more than two-fold alteration in transcription level was taken into consideration for each gene probe. For diamide and H_2_O_2_, all recovery phase (3–9 h of exposure) data were disregarded. Letters D, H and M stand for diamide, H_2_O_2 _and menadione, respectively. When gene probes responsive to more than one agent were counted {D and H and M (DHM), H and M (HM), D and H (DH), D and M (DM)} only gene probes affected by the selected agents in the same direction, *i.e*. were equally up-regulated or equally down-regulated, were considered.

In total, 2499 gene probes (available in Additional file 2:Supplement2 for the list of oxidative stress responsive gene probes) were affected by at least one of the stress-inducing compounds tested. Prior to gene distribution analysis, recovery phase diamide (3–6 h) and H_2_O_2 _(3–9 h) data were disregarded, and only gene probes with at least 60 % of the M' values available for each stress-inducing agent (diamide: 15 min – 1 h, H_2_O_2_: 15 min – 1 h, menadione: 30 min – 9 h) were processed further resulting in1502 gene probes in total (Figure 5, Additional file 2:Supplement2 for the list of oxidative stress responsive gene probes). Half (52.7 %) of the selected gene probes responded to more than one agent, 35.1 % were influenced by the agents equally – up- or down-regulated, while 17.6 % of them were effected differentially. The number of gene probes affected by stress increased in the order of H_2_O_2 _(42.0 %), diamide (49.2 %) and menadione (81.6 %). Similar tendencies were observed for gene probes affected only by one agent, H_2_O_2 _(6.3 %), diamide (8.4 %) and menadione (32.6 %). There were large sets of transcripts within the genome that were concomitantly responsive to several agents, e.g. diamide and H_2_O_2 _and menadione (20.2 %); H_2_O_2 _and menadione (11.9 %); diamide and H_2_O_2 _(3.7 %), and diamide and menadione (17.0 %) (Figure 5, Additional file 2:Supplement2 for the list of oxidative stress responsive gene probes). After filtering out data where the agents caused more than two-fold but opposite changes in transcription the ratios of diamide-H_2_O_2_-menadione, H_2_O_2_-menadione, diamide-H_2_O_2 _and diamide-menadione responsive gene probes went down to 13.0, 7.7, 2.6 and 11.8 %, respectively (Figure 5).

**Figure 6:**
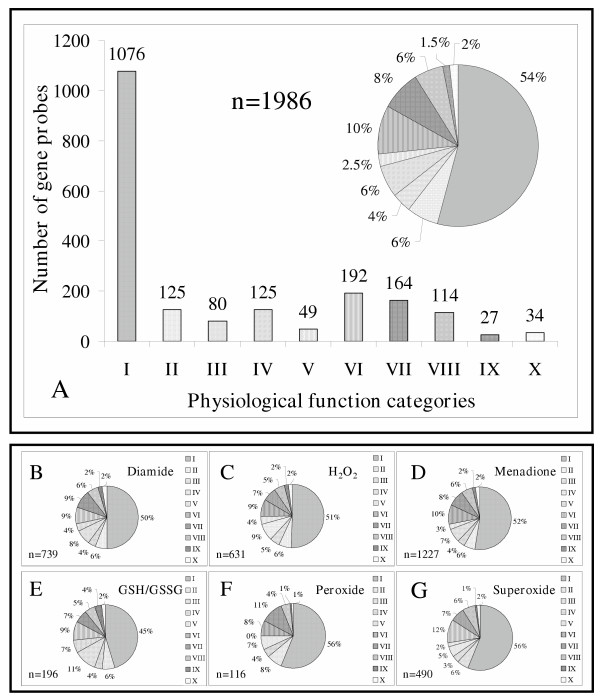
Distribution of selected gene probes among physiological function categories. Gene probes were chosen using the 60 % data availability criteria and disregarding recovery phase changes for diamide and H_2_O_2_. **Part A**. Functional distribution of gene probes (1986 in total; Additional file 3:Supplement3 for the list of gene probes considered in significant enrichment calculations) considered in significant enrichment calculations (Figure 7). **Parts B-G. **Distribution of gene probes as a function of physiological function categories as well as stress-inducing agents (**Parts B-D**) and oxidative stress-responsive physiological parameters (**Parts E-G**). For these analyses, gene probes influenced by treatments (**Parts B-D**) or responsive to changes in physiological parameters (**Parts E-G**) were selected; n values stand for the gene probes involved in separate functional distribution analyses. Gene probes equally responsive to dimide, H_2_O_2_, menadione treatments, or to H_2_O_2 _and menadione but not to diamide or solely responsive to menadione were regarded as GSH/GSSG, O_2_^2- ^or O_2_^•-^-responsive, respectively. Segments of the diagrams represent the following physiological function categories: I, "Function-unknown"; II, "Signal generation and transduction, DNA transcription, regulation"; III, "Replication, cell division cycle and development"; IV, "RNA splicing and translation, protein maturation"; V, "Defense and stress proteins, degradation of xenobiotics; VI, "Transport, cytoskeleton, cell wall"; VII, "Carbon metabolism"; VIII, "Nitrogen and sulfur metabolism"; IX, "Secondary metabolism"; X, "Oxidoreductases".

In order to perform gene enrichment calculations, the group of stress-responsive gene probes (1502; Additional file 2:Supplement2 for the list of oxidative stress responsive gene probes) was supplemented with a set of gene probes not influenced by oxidative stress but obeying the 60 % data availability filtering criteria (484 in total). The combined set of gene probes (1986 in total; Figure 6A, Additional file 3:Supplement3 for the list of gene probes considered in significant enrichment calculations) was used for functional data sorting and statistical analysis. Stress-responsive gene probes were recorded in highest numbers in categories "Transport, cytoskeleton, cell wall", "Carbon metabolism" and "RNA splicing and translation, protein maturation". The ratio of "Function-unknown" genes was around 50 % independently of stress-inducing agent (Figures 6B–D). The distribution of stress-responsive gene-probes among functional categories was approximately the same for each stress-inducing agent (Figures 6B–D). When GSH/GSSG (gene probes equally influenced by diamide, H_2_O_2 _and menadione treatments), peroxide (gene probes equally influenced by H_2_O_2 _and menadione treatments) and superoxide (solely influenced by menadione treatment) responsive gene probes were selected, the distribution of gene probes among functional categories was distinct (Figure 6E–G).

**Figure 7:**
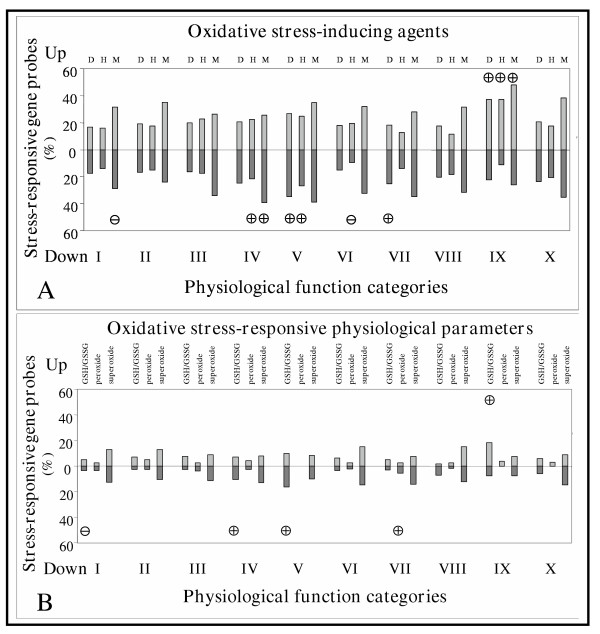
Significant gene enrichment profiling. The proportions of stress responsive gene probes influenced by diamide (D), H_2_O_2 _(H) and menadione (M) treatments are shown in each physiological function category in **Part A**. The ratios of GSH/GSSG, peroxide and superoxide responsive gene probes calculated for each physiological function category are presented in **Part B**. Gene probes influenced by dimide, H_2_O_2_, menadione treatments in the same way, or by H_2_O_2 _and menadione but not by diamide or solely responsive to menadione were regarded as GSH/GSSG, O_2_^2- ^or O_2_^•-^-responsive, respectively. Symbols ⊕ and  indicate significant overrepresentation and underrepresentation of stress-responsive gene probes, respectively, in separate physiological function categories. *p *values and significant enrichments recorded in sets of gene probes not responding to oxidative stress triggered by diamide, H_2_O_2 _and menadione treatments are presented in Additional file 4:Supplement4 for the results of significant enrichment calculations.

As shown in Figure 7A and 7B and in Additional file 4:Supplement4 for the results of significant enrichment calculations, oxidative stress responsive gene probes were significantly overrepresented among diamide, H_2_O_2 _and menadione induced "Secondary metabolism", diamide repressed "Defense and stress proteins, degradation of xenobiotics" and "Carbon metabolism", H_2_O_2 _repressed "RNA splicing and translation, protein maturation" and "Defense and stress proteins, degradation of xenobiotics" and menadione repressed "RNA splicing and translation, protein maturation" gene probes. Moreover, significant underrepresentation of stress-responsive gene probes was observed in categories such as "Function-unknown" and "Transport, cytoskeleton, cell wall" among menadione and H_2_O_2 _repressed gene probes, respectively. When enrichment calculations were performed on GSH/GSSG, peroxide and superoxide responsive gene probes, a different enrichment profile was found with gene probes enriched in functional categories such as "RNA splicing and translation, protein maturation" (GSH/GSSG repressed probes), "Defense and stress proteins, degradation of xenobiotics" (GSH/GSSG repressed probes), "Carbon metabolism" (peroxide repressed probes), "Secondary metabolism" (GSH/GSSG induced probes) and underrepresented among GSH/GSSG repressed "Function-unknown" genes.

Finally, in addition to Additional file 2:Supplement2, for the list of oxidative stress responsive gene probes, the full list of oxidative stress responsive gene probes is available at National Center for Biotechnology Information (NCBI) Gene Expression Omnibus (GEO) [13] on Platforms GPL1752 and GPL1756. Oxidative stress sensitive genes equally responsive to diamide, H_2_O_2 _and menadione treatments, i.e. the likely GSH/GSSG-responsive genes, are briefly summarized in Additional file 5:Supplement5 for a selection of GSH/GSSG responsive genes. A selection of genes considered as ROS (superoxide or peroxide) responsive is given in Additional file 6:Supplement6 for a selection of ROS responsive genes.

## Discussion

ROS-generating chemicals are frequently used as agents to induce antioxidative defense systems and to study the mechanism of adaptation to oxidative stress [14]. Although organisms might respond to naturally occurring oxidative stress conditions [15] or endogenous ROS [3] in a quite different way these reactants are highly suitable to optimize experimental conditions and, hence, to improve the reproducibility of the size and intensity of stress sensed by cells [6,7]. Without fine-tuning experimental conditions, e.g. optimizing the quantities of oxidant added, these chemicals have been shown to affect the concentrations of multiple types of ROS and trigger concomitantly severe GSH/GSSG redox imbalance [16].

Among the oxidative stress-inducing agents selected, diamide is a thiol-oxidizing agent resulting in fast oxidation of GSH to GSSG resulting in GSH/GSSG redox imbalance [18], H_2_O_2 _increases intracellular peroxide (O_2_^2-^) levels, which leads to the direct oxidation of the sulfur-containing amino acids and the generation of OH^• ^radicals [18], menadione generates superoxide anions (O_2_^•-^) in a redox cycle, which destroys 4Fe-4S proteins productng of OH^• ^[18]. Menadione may also affect GSH pool directly *via *a detoxification reaction catalyzed by GST [15].

Due to distinct mechanism of action, these chemicals provide the opportunity to map GSH/GSSG, O_2_^2- ^and O_2_^•-^-responsive sets of genes within the *A. nidulans *genome. In yeast, there is literature supporting the assumption that these agents influence different elements of the oxidative stress response [5,19-29].

In the β-lactam producer *P. chrysogenum*, diamide, H_2_O_2_, *tert*-butyl hydroperoxide and menadione were used to set up systems where intracellular GSH/GSSG redox balance as well as intracellular peroxide and superoxide levels were altered selectively without influencing the other two physiological parameters [6,7].

Unfortunately, such clear-cut experimental conditions could only be set up using diamide in *A. nidulans*, which, when employed in a concentration of 1.8 mM, disturbed only GSH/GSSG redox balances without increasing intracellular ROS concentrations (Figure 1, Additional file 1:Supplement1 for physiological changes). H_2_O_2 _and menadione always affected the GSH/GSSG redox state of the cells, and menadione also influenced intracellular peroxide levels in addition to superoxide concentrations (Figure 1, Additional file 1:Supplement1 for physiological changes). This can be explained with the 6-17-fold lower specific catalase activities detected in stress-exposed *A. nidulans *cells in comparison to *P. chrysogenum *cultures (Additional file 1 :Supplement1 for physiological changes) [7]. The relatively weak peroxide-decomposing capability of *A. nidulans *explains the disturbance of GSH/GSSG redox balance with H_2_O_2 _and menadione, because GSH is also oxidized non-enzymatically by many ROS including OH^•^, a free radical known to be generated under these treatments [14,18].

Because H_2_O_2 _and menadione influenced the GSH/GSSG redox couple and intracellular ROS levels in an inseparable manner 0.8 mM and 75 mM concentrations were selected, respectively, for DNA microarray experiments taking into consideration cell survival and gene expression data (Additional file 1:Supplement1 for physiological changes, Figure 2). The specific activities of selected antioxidative enzymes were increased (Additional file 1:Supplement1 for physiological changes) and cell survival rates were not affected significantly by them (data not shown), and no deterioration of the mRNA and rRNA populations were observed under these conditions (Figure 2A). Stress-inducing agents employed in these selected concentrations triggered detectable transcriptional changes in cDNA microarray experiments as visualized on Northern blots (Figure 2A).

Similarly to *P. chrysogenum *[6,7], there was a correlation between intracellular GSH/GSSG values, ROS levels and increase in specific antioxidative enzyme activities in *A. nidulans *(Additional file 1:Supplement1 for physiological changes). For example, diamide failed to induce SOD activity (O_2_^•-^-responsive) and H_2_O_2 _did not influence SOD (O_2_^•-^-responsive) and GST (catalyses GSH-dependent reaction) activities after 1 h exposure (Additional file 1:Supplement1 for physiological changes). As shown in Figure 2, the selected stress-inducing agent concentrations also allowed us to record ROS and GSH/GSSG specific differences at the level of gene transcripts.

Normalized DNA microarray data correlated with transcriptional changes determined on Northern blots (Figure 3). Because the calculation of both M and M_Northern _values included division of gene expression data recorded in stress-exposed cultures with their counterparts found in controls the non-stress-related incubation-time-dependent changes taking place in control cultures distorted these parameters and, hence, disturbed their correlation especially at longer incubation periods (Figures 2C, 2D, 2F and 2G). M' = f(t) functions for individual gene probes were fluctuating in many cases but these tendencies might represent either real fluctuations like in the case of the homologue of the Ca^2+^-calmodulin-dependent serine-threonine-protein kinase (Figures 2A and 2G) or virtual fluctuations – a result of non-stress-related changes in controls (Figure 2C). Taking into consideration the high frequency of fluctuating gene expression patterns we considered only transcriptional changes above or below the [+1;-1] M' thresholds as a function of incubation time during gene function analysis (Figure 6, Additional file 2:Supplement2 for the list of oxidative stress responsive gene probes, Additional file 3:Supplement3 for the list of gene probes considered in significant enrichment calculations, Additional file 5:Supplement5 for a selection of GSH/GSSG responsive genes, Additional file 6:Supplement6 for a selection of ROS responsive genes).

All three chemicals used to trigger oxidative stress induced a genome-wide transcriptional stress response, which reached its maximum at 1 h incubation time as indicated by the number of gene probes affected more than two-fold by oxidative stress (Figure 4). The induction of antioxidative enzymes by H_2_O_2 _is regarded in general faster than that triggered by menadione as exemplified by the transcriptional changes recorded for Cu,Zn-containing superoxide dismutase in the yeast *S. pombe *[27] and for the *sodA *gene in this study (more than two-fold up-regulation was observable between 3–6 h incubations in menadione-treated cultures, Figures 2A, 2C and 2F). In agreement with this, the kinetics of intracellular accumulation of ROS and decrease in GSH/GSSG redox ratio was slow although steady in the presence of 0.8 mM menadione (Figure 1). Nevertheless, up-regulation of *gstA*, which plays a predominant role in detoxification of menadione [15], as early as 30 min (Figure 3A) was indicative of an early influx of this agent into cells. Menadione is also an effective arylating agent in addition to its redox-cycling behavior and, especially at high concentrations (higher than 0.2 mM), it enhances membrane fluidity [30]. Oxidative and non-oxidative cell injuries caused by menadione are likely to contribute to short-term but large-scale transcriptional response observed in menadione-treated cultures (Figure 4). The transient decrease in the ratio of stress-affected gene-probes at 3–6 h exposure times and the appearance of a second maximum at 9 h (Figure 4) may be indicative of a shift from a mixed oxidative/non-oxidative towards a pure oxidative stress response in the gene expression signature of menadione.

Diamide, H_2_O_2 _and menadione all influenced large groups of genes within the genome with considerable overlap (Figures 5 and 6). The appearance of large groups of gene probes equally responsive to diamide, H_2_O_2_, menadione (likely GSH/GSSG-dependent regulation), or to H_2_O_2 _and menadione but not to diamide (likely O_2_^2-^-dependent regulation) or solely responsive to menadione (likely O_2_^•-^-dependent regulation) highly supported the hypothesis that separate ROS and GSH/GSSG redox state regulated groups of genes are present in a genome. The ratios of O_2_^2-^-, O_2_^•-^- and GSH/GSSG responsive genes were estimated at 7.7, 32.6 and 13.0 %, respectively (Figure 5).

The ratio of O_2_^•-^-responsive gene probes was likely overestimated to an unknown extent by counting genes that were induced or repressed exclusively by non-oxidative types of stress also triggered by 0.8 mM menadione [30]. Moreover, one must consider that the oxidative stress-inducing agents used in this study may also disturb physiological parameters other than O_2_^•-^, O_2_^2-^, GSH and GSSG concentrations, e.g. the production of lipid peroxides [31,32], and these changes may also have significant impact on global gene expression patterns as indicated by the relatively high ratios of only H_2_O_2 _(6.3 %), only diamide (8.4 %), diamide and menadione and but not H_2_O_2 _(11.8 %; e.g. *gstA*; Figures 2B and 2E, Additional file 2:Supplement2 for the list of oxidative stress responsive gene probes) and H_2_O_2 _and diamide but not menadione (2.6 %)responsive gene probes (Figure 5). In *S. cerevisiae*, cells exposed to the toxic lipid peroxidation product linoleic acid hydroperoxide (LoaOOH), a distinct set of genes was stimulated that were not responsive to other oxidants or heat shock [32]. The frequent appearance of gene probes responding to different stress-inducing agents with a more than two-fold change in transcription but in the opposite way (17.6 % in total) may also be the consequence of the effects of either not-yet-identified physiological factors or non-systematic errors of DNA microarray hybridizations. All these factors may further distort the ratios calculated for O_2_^2-^-, O_2_^•-^- and GSH/GSSG-responsive gene probes as well.

Gene probe distribution analysis (Figures 6 and 7) and significant enrichment calculations (Figure 7, Additional file 3:Supplement3 for the list of gene probes considered in significant enrichment calculations, Additional file 4:Supplement4 for the results of significant enrichment calculations) indicated that the distribution of stress responsive gene probes between functional categories and their enrichment profiles were different and only partially overlapping when grouped according to their responsiveness to stress-inducing agents or to changes in physiological parameters. This finding indicates that genome-wide transcriptional changes should be analyzed by correlating them directly to changes in oxidative stress-response physiological parameters and not to the chemistry and concentration of oxidant used to trigger stress.

According to Shafer and Buettner [1], the GSH/GSSG redox couple plays a pivotal role in controlling thiol and disulfide nano-switches that move cells from proliferation, through various stages of differentiation and, when the redox environment of the cells cannot be maintained, towards apoptosis. In accordance with this, our observations reflect the distinguished role of the GSH/GSSG redox state in genome-wide oxidative stress response (Additional file 5:Supplement5 for a selection of GSH/GSSG responsive genes). For example, many components of signal transduction pathways, including homologues of PBS2 like MAPK kinase (related to the *S. cerevisiae *PBS2 and the *S. pombe *Wis1 MAPK kinases, which phosphorylate HOG1 and Spc1-Sty1 MAPKs, respectively) [33], PSK2 kinase [34], important transcription factors (e.g. AtfA, a homologue of the *S. pombe *Atf1 [27,35,36]), ubiquitin tagging, cell cycle regulators, translation machinery proteins, defense and stress proteins (e.g. glutathione peroxidase, trehalose synthase), transport proteins (e.g. P-type Na^+^-ATPase) as well as many enzymes of the primary (e.g. glycolytic enzymes) and secondary (e.g. sterigmatocystin biosynthetic enzymes) metabolism, seemed to be responsive to GSH/GSSG redox imbalances at the level of transcription (Additional file 5:Supplement5 for a selection of GSH/GSSG responsive genes).

The stress-activated protein kinase (SAPK) signal transduction pathway (MAPK Spc1-Sty1-dependent signal transduction) and the bZip transcriptional factor Atf1 have been shown to play a pivotal role in the regulation of the core environmental stress response (CESR) in *S. pombe*, which is common to all stresses including oxidative stress [37]. The stress-responsive genes controlled by Atf1 in *S. pombe *include e.g. *gpx1 *glutathione peroxidase (oxidative, osmotic and thermal stress) [35], *tps1 *trehalose-6-phosphate synthase (oxidative and salt-induced osmotic stress) [36] and *cta3 *cation-transporting P-type ATPase (salt-induced osmotic stress) [38], and *atf1 *itself is an induced CESR gene [37]. Based on functional and sequence homologies as well as gene expression changes presented in Additional file 5:Supplement5 for a selection of GSH/GSSG responsive genes, the existence of an oxidative stress responsive SAPK-AtfA regulatory pathway with a similar set of target genes is likely in *A. nidulans *as hypothesized by Aguirre et al. [3]. But this pathway seems to be controlled primarily by changes in the GSH/GSSG redox balance instead of ROS (peroxide) concentrations [3].

The GSH/GSSG-dependent down-regulation of ribosomal proteins located in the cytoplasm of oxidative stress-exposed *A. nidulans *cells (Additional file 5:Supplement5 for a selection of GSH/GSSG responsive genes) may result in a decrease in the rate of protein synthesis similar to LoaOOH-exposed *S. cerevisiae *cells [32]. Genes encoding ribosomal proteins located in the mitochondria was induced (Additional file 5:Supplement5 for a selection of GSH/GSSG responsive genes) indicating an increased protein synthesis in these organelles.

GSH/GSSG redox imbalances affect glycolysis at 6-phosphofructo-2-kinase (induction) and fructose biphosphate aldolase (repression), which may result in the intracellular accumulation of fructose-1,6-bisphosphate. This glycolytic metabolite is known to protect cells by improving energy status and mitochondrial functions in mammals [39,40]. In accordance with the findings published by Jayashree and Subramanyam [31], the synthesis of the mycotoxin sterigmatocystin (a precursor of aflatoxin B_1 _in aflatoxin-producing fungi) is regulated positively by oxidative stress (Additional file 5:Supplement5 for a selection of GSH/GSSG responsive genes, Figure 7).

Large gene groups responsive to ROS but not to changes in the GSH/GSSG redox balance (Additional file 6:Supplement6 for a selection of ROS responsive genes) indicated that GSH-independent stress-sensing regulatory network(s) also operated in *A. nidulans *cells exposed to oxidative stress. O_2_^2- ^and O_2_^•- ^species affected mainly negatively transport processes like nuclear export-import *via *the down-regulation of the *ran/spi1 *homologue [41,42] and *ntf2 *nuclear transport factor 2 [42,43] and the induction of the yeast *GTR2 *homologue, a negative regulator of the Ran G protein cycle [44] (Additional file 6:Supplement6 for a selection of ROS responsive genes). ROS also seem to influence signal transduction e.g. *via*. repression of the homologue of the *Cryphonectria parasitica *cppk1 protein kinase [45]. At the level of transcription, CpcA-JlbA amino acid starvation-responsive transcription factors [46,47] are likely to contribute substantially to the regulation of oxidative stress response against ROS (Additional file 6:Supplement6 for a selection of ROS responsive genes). Opposite to GSH/GSSG-dependent regulation (Additional file 5:Supplement5 for a selection of GSH/GSSG responsive genes), translation was hardly effected by ROS but the degradation of abnormal or damaged proteins in mitochondria by Lon protease [48] was induced by superoxide. Elements of sexual development and sporulation were found to be ROS-responsive, including a homologue of the meiosis inducer kinase IME2 (yeast) [49,50], AspC septin, SpoC1-C1C conidium-specific protein [51] and a homologue of the cdc5 kinase [52,53] (Additional file 6:Supplement6 for a selection of ROS responsive genes). The down-regulation of the latter kinase together with a blockage in the nuclear export-import system [41-44] and a superoxide-dependent repression of the *benA *hyphal tubulin gene [54] may lead to a cell cycle arrest in oxidative stress. H_2_O_2 _and menadione have been reported to cause G_2 _and G_1 _phase cell cycle arrests in *S. cerevisiae*, respectively [55], and several cell-cycle-regulated transcripts were also shown to be down-regulated in linoleic acid hydroperoxide-exposed *S. cerevisiae *cells [32].

In addition to the harmful effects on living cells, there is a growing appreciation of ROS as important signal molecules in diverse cellular processes [1,4]. For example, ROS (superoxide, H_2_O_2_) generated endogenously by NADPH oxidases (NOXs) have been demonstrated to play a role in macrophage [56,57] and insulin [58] signaling and regulation of vascular functions [4,59] in mammals. NOX-generated ROS also regulates cell expansion in the plant *Arabidopsis thaliana *[60]. NOX-generated ROS have also been shown to regulate the sexual development in *A. nidulans *[3,61] and *Podospora anserina *[62] as well as ascospore germination in *P. anserina *[62] and *N. crassa *[3], which clearly points that ROS are likely involved in regulation of development and cell physiology in filamentous fungi as well.

## Conclusion

Large and separate ROS (O_2_^2-^, O_2_^•-^) and GSH/GSSG redox groups of genes are present in the *A. nidulans *genome. ROS and GSH/GSSG signaling seems to proceed independently of each other and may express their effects *via *different regulatory networks. Unless the experimental conditions are optimized oxidative stress-inducing agents are likely to influence many physiological parameters and a concomitant activation of these separate signal transduction and regulatory networks can be predicted to reach an appropriate stress response at the level of transcriptome.

Considering that diamide – GSH/GSSG, H_2_O_2 _– O_2_^2- ^and menadione – O_2_^•- ^responsive sets of genes were only partly overlapping and their significant enrichment profiles in functional categories, we recommend that oxidative-stress-triggered global transcriptional changes should be evaluated by correlating them to changes in oxidative-stress-responsive physiological parameters. Correlating genome-wide transcriptional changes directly to the chemistry and concentrations of stress-inducing agents might lead to unsatisfactory conclusions unless the experimental conditions are thoroughly optimized and physiologically characterized.

DNA microarray data presented in this study demonstrate changes in the transcriptome of oxidative stress-exposed *A. nidulans *cells. A more direct connection of genome-wide transcriptional changes to cellular physiology will be reached by a thorough and integrative analysis of stress-provoked changes observable at the levels of proteome and metabolome [63], which is now in progress in our laboratory.

## Methods

### Strain, culture conditions, cellular physiology

*A. nidulans *FGSC 26 (*biA1*, *veA1*) was grown in shake flasks (500 ml) containing 100 ml minimal-nitrate medium, pH 6.5 [64] supplemented with 0.5 % yeast extract. Culture media were inoculated with 1 × 10^8 ^spores and were incubated for 18 h at 37°C and 3.3 Hz shaking frequency. Mycelia were separated by filtration on sintered glass and were transferred immediately into pre-incubated 100 ml aliquots of minimal-nitrate medium, pH 6.5 either supplemented with stress-inducing agents (stress-exposed cultures) or left untreated (controls).

"*Dosis lethalis minima*" (DLM) values for diamide (azodicarboxylic acid *bis *[*N*,*N*-dimethylamide]), H_2_O_2 _and menadione (menadione sodium bisulfite, a water-soluble derivative of 2-methyl-1,4-naphthoquinone was used in all experiments [24]) were determined in the minimal-nitrate medium, supplemented with the oxidative stress-inducing agents in different concentrations (diamide: 1.0–3.0 mM; H_2_O_2_: 50–750 mM; menadione: 0.2–2.0 mM) and measuring cell survivals after 9 h treatments (37°C, 3.3 Hz shaking frequency). Cell survival rates were estimated after transferring stress-exposed mycelia into 100 ml aliquots of oxidant-free minimal-nitrate medium by monitoring the increase in the dry cell mass (DCM) for 24 hours (37°C, 3.3 Hz shaking frequency) [6,7]. DLM was defined as the lowest oxidant concentration that resulted in a complete lack of growth of treated mycelia in the oxidant-free medium.

Changes in intracellular concentrations of reactive oxygen species (ROS; O_2_^•-^, O_2_^2-^) and GSH/GSSG balance were optimized to keep the number of physiological parameters affected by either diamide, H_2_O_2 _or menadione triggered stress as low as possible [6,7]. This goal was approached by exposing 18 h mycelia to these agents dissolved at several concentrations in the minimal-nitrate medium. Intracellular GSH, GSSG, peroxide and superoxide levels were determined at 15 min, 30 min, 1 h, 3 h, 6 h and 9 h exposure times.

For GSH and GSSG determinations, mycelia from 5–10 ml aliquots of both stress-exposed and control cultures were filtered out and washed with ice-cold sterile distilled water. Mycelial mats were re-suspended in ice-cold 5 % (w/v) 5-sulfosalicylic acid by vigorous mixing and left for 20 min at 0°C [7]. After centrifugation at 10,000 g for 10 min, the supernatants were neutralized with triethanolamine at 0°C. The specific intracellular GSH and GSSG levels were determined as described elsewhere [6,7,65].

The intracellular peroxide and superoxide levels were estimated in separate experiments by monitoring the formation of 2',7'-dichlorofluorescein (DCF) from 2',7'-dichlorofluorescin diacetate and ethidium (Et) from dihydroethidium, respectively, as described before [7,66].

Changes in the specific activities of selected antioxidative enzymes were also measured in separate experiments. In these cases, 18 h mycelia were treated with oxidative stress-inducing agents in minimal-nitrate medium for 1 or 6 h. Stress-exposed mycelia were harvested by filtration on sintered glass, were washed with and re-suspended ad frozen immediately in ice-cold 0.1 M K-phosphate buffer (pH 7.5). Cell-free extracts were prepared by X-pressing and centrifugation [67]. Specific glutathione *S*-transferase (GST) [68], catalase [69] and superoxide dismutase (SOD) [70] activities were measured according to the literature shown in parentheses.

DCM was determined as described elsewhere [71], and protein contents of the cell-free extracts were measured by a modification of the Lowry method [72].

### RNA extraction and Northern blot hybridization

Mycelia from both stress-exposed and control cultures (15 min, 30 min, 1 h, 3 h, 6 h and 9 h cultivation times) were filtered out, washed with ice-cold sterile distilled water, transferred immediately into pre-cooled (-70°C) Eppendorf tubes, were frozen at -70°C and, subsequently, were vacuum-dried overnight at room temperature. Total RNA was extracted from approximately 100 mg quantities of dried mycelial mats using TRISOL reagent (Invitrogen) as recommended by Chomczynski [73]. Crude RNA preparations were dissolved in 300 μl DEPC-treated water by agitation with sterile pipette tips at 68°C for 20–30 min. The samples were cooled down on ice, and RNA pools were precipitated with 300 μl 4.0 M LiCl (prepared in 20 mM Tris-HCl pH = 7.5 buffer, also containing 10 mM EDTA) and were left at -20°C overnight. RNA pellets were collected with centrifugation (12000 g, 30 min, 4°C) and were washed twice with pre-cooled (-20°C) 75 % ethanol. RNA preparations were air-dried and immediately re-suspended in 50 μl aliquots of diethyl pyrocarbonate (DEPC)-treated water. RNA content was always estimated in 300-fold diluted samples by measuring the absorbance at λ = 260 nm. The purity of the preparations was estimated by calculating A_260_/A_280 _absorbance ratios, which were always approximately 1.8. RNA preparations were stored at -80°C in 10 μl aliquots to avoid repeated freezing and thawing. The quality of RNA preparations was also checked by running 7.5 μg quantities of RNA in 1.2 % agarose gel.

Electrophoreses and Northern blot analyses were performed as described elsewhere [74-76]. Briefly, 7.5 μg quantities of refined RNA samples were separated in 1.2 % agarose gels containing 3 % formaldehyde and were blotted onto Hybond-N^+ ^(Amersham) membranes. RNA molecular weight calibration was performed using Invitrogen 0.24–9.5 Kb RNA Ladders as standards according to the protocol of the manufacturer. Occasionally, the blots were re-used after stripping them in boiling water in the presence of 0.1 % SDS [10].

Probes for Northern hybridization were prepared by PCR-amplification of specific sequences for *aoxA *(mitochondrial alternative oxidase), *gstA *(glutathione *S*-transferase), *sodA *(Cu,Zn-superoxide dismutase precursor), *sconC *(sulfur metabolic regulator) and ORF AN4483.2 (a close homologue of *Schizosaccharomyces pombe *Ca^2+^-calmodulin-dependent serine-threonine-protein kinase) using custom-made cDNA plasmid libraries (Life Technologies) [10]. The oligonucleotide PCR primers employed in this study were: *aoxA*, F: 5'-TAG CCG ACG AAA CGA TGA CAG GTA-3' and R: 5'-CCT GCT CCG ATG GGG TAT GGA-3'; *gstA*, F: 5'-GGT AAC GCC TGC TCT TAG GCT A-3' and R: 5'-ATT TCG AGC TCA ACT GAC TGC A-3'; *sodA*, F: 5'-ATG CGC TCA ACC CTT ACG CAA-3' and R: 5'-CTT TAC CCG CGG CAA GGC TA-3'; *sconC*, F: 5'-TAA CAA GCA AGA CGA AAC CGC TGA-3' and R: 5'-AAG GGC AAG TCC CCC AGG A-3'; ORF AN4483.2, F: 5'-TTA TGC GCC AGA TCG ATC ACC CTA-3' and R: 5'-GCC AAC ATC AAA GAC TTC ACG CAA-3', all of them designed by the GeneFisher [77] software. The PCR program included the following steps: 96°C for 3.0 min, 94°C for 0.30 min, t = T_melting_-2.0°C for 0.45 min, 72°C for 1.30 min, amplification cycle repeated 29 times, 72°C for 10.0 min, 15°C for 1 h. To calculate the size of amplified DNA fragments Invitrogen Low DNA Mass Ladder (100–2000 bp) was used in 0.8 % agarose gels prepared in 1 × TAE buffer. Expected and found sizes for cDNA amplificants were always in good agreement.

cDNA fragments were extracted from the 0.8 % agarose gels and purified using QIAEX II Gel Extraction Kit (Qiagen) as recommended by the manufacturer. The DNA preparations were always re-dissolved in ion-exchanged H_2_O. The efficiency and yield of DNA extraction was always checked by repeated run on 0.8 % agarose gels. ^32^P-labelled probes were prepared using Invitrogen RadPrime DNA Labelling System according to the manufacturer's protocol employing 25 ng quantities of purified cDNA fragments and 50 μCi (3.3 mM; Amersham Biosciences) ^32^PdCTP.

Hybridizations were carried out in Church buffer in rolling chambers at 62°C overnight [75,76,78]. Membranes were washed once with 2 × SSC/0.2 % SDS and once with 0.1 × SSC/0.1 % SDS solutions at 62°C for 1 h, and were subsequently exposed to Kodak XAR-5 films at -80 C for 2–5 days. Optical densities of autoradiographic bands were recorded with a Model GS-700 Imaging Densitometer (Bio-Rad) and were analyzed with a combined Bio-Rad Gel Doc 1000 and Mitsubishi Video Copy Processor system.

### DNA microarray hybridization

The DNA microarrays employed throughout this work contained 3533 unique PCR-amplified probes, identified as first generation in previous publications and printed in 4073 spots, which were duplicated [8,9]. The full description of gene probes including PCR primer(s), Oklahoma State University contig IDs (OSU contig IDs) [79] and Broad Institute (Cambridge, MA, USA) ORF IDs [80] are given at NCBI GEO [13] on Platforms GPL1752 and GPL1756.

Changes in cDNA populations, prepared from oxidative stress exposed and untreated control cultures, were estimated with Genisphere (Hatfield, PA, USA) 3DNA Submicro EX Expression Array Detection Kit. For each hybridization, 60 μg of total RNA pooled from three biological samples (20 μg each) were used for cDNA synthesis using SuperScript II Reverse Transcriptase Enzyme (Invitrogen). Hybridizations where performed as recommended by Genisphere using the SDS-based hybridization buffer provided with 1.0 μl denaturant COT-1 DNA (10 μg ml^-1^, GIBCO 15632-011) added, Lifter Slip cover slip (Erie Scientific Co., Portsmouth, NH, USA) and CMT-Hybridization Chamber (Corning Incorporated Life Sciences, Corning, NY, USA) at 65°C. Following hybridization, spot intensities were determined using a GenePix 4000 B microarray scanner (Axon Instruments).

### Data normalization and statistics

In DNA microarray experiments, means, medians and standard deviations (S.D.s) for both dyes and for both spot and background intensities were calculated with GenePix Pro 3.0 software. Defected spots with false readings were filtered out manually and were disregarded in further calculations. We also filtered out and omitted those data points where background mean plus1 S.D. was higher than the spot intensity mean for both dyes. After data filtering, the background-corrected ratios and log_2 _ratios (M) of spot intensities were calculated. Block-by-block LOESS normalization was then applied [81] using SAS for Windows, version 8 (SAS Institute Inc., Cary, NC, USA.) software package. In further data processing, normalized spot intensity ratios (M') were analyzed.

The effects of different stress-inducing agents on global gene expression patterns were quantified by counting the gene probes responding to the treatments above or below the [+1;-1] M' thresholds ('two-fold rule') [82]. Gene probes affected by more than one agent were also screened. In the latter case, only the first more than two-fold change in gene expression was taken into consideration for each gene probe disregarding all gene probes that responded to different agents oppositely.

Correlation between DNA microarray M' and appropriate M = log_2_(optical density_stress-exposed_*optical density_control_^-1^) values for Northern blot autoradiography pictures was calculated with Microsoft Excel 97 software.

In physiological optimization of experimental conditions, the variations between experiments were estimated by standard deviations (S.D.) and the statistical significance of changes in physiological parameters was estimated by the Student's *t*-test [6,7]. Only the probability levels of *P *≤ 5 % were regarded as indicative of statistical significance.

### Homology search, data mining and significance enrichment calculations

EST sequence processing and functional data sorting were performed using PipeOnline version 2.0 [79,83]. Homology search was carried out in two independent ways: (i) *via *translated contig sequence query *versus *protein in NCBI BLAST (blastx) [84] and (ii) *via *translated ORF (Broad Institute, Cambridge, MA, USA) [80] query *versus *protein in NCBI BLAST (blastp) [84]. In data sorting, only homologies with Expect value lower than E-40 were considered [85]. Physiological function categories were defined according to previous literature data on transcriptional changes observable under oxidative and other kinds of environmental stress in fungi and PipeOnline functional data sorting based on NCBI protein records and MPW Metabolic Pathways Database functional dictionary [79,83] (release date December 2002). Importantly, each gene probe was characterized with one annotated function.

For significant enrichment calculations, the hypergeometic distribution test was carried out [86,87] for selected groups of genes using SAS for Windows, version 8 (SAS Institute Inc., Cary, NC, USA.) software.

## List of abbreviations

CESR – core environmental stress response

DCM – dry cell mass

DCF – 2',7'-dichlorofluorescein

DEPC – diethyl pyrocarbonate

DLM – *dosis lethalis minima*

EST – expressed sequence tag

Et – ethidium

FUN – function-unknown

GSH – glutathione

GSSG – glutathione disulfide

GST – glutathione *S*-transferase

LoaOOH – linoleic acid hydroperoxide

MAPK – mitogen-activated protein kinase

NOX – NADPH oxidase

OSU – Oklahoma State University

ROS – reactive oxygen species

SAPK – stress-activated protein kinase

SOD – superoxide dismutase

## Authors' contributions

IP and MM carried out DNA chip hybridizations, filtered microarray data and performed homology search. ZK and PA processed and normalized DNA microarray data. NCBI GEO data base and Additional file 2:Supplement2 for the list of oxidative stress responsive gene probes were constructed by MM, ZK and PA. Optimization of experimental conditions and enzyme assays were done by ET and TP. Northern hybridizations and evaluation of Northern data were performed by IP. GB and RAP contributed crucially to the conception and design of the experiments presented in this paper as well as to the discussion of DNA microarray data and the outcomes of homology search. IP was a Fulbright Research Fellow at OSU meanwhile his work was supervised by RAP.

## Supplementary Material

Additional File 1 Physiological changes recorded at selected dimide, H_2_O_2 _and menadione concentrations and exposure times. DLM values and concentrations selected for DNA microarray experiments are shown for diamide, H_2_O_2 _and menadione together with specific SOD, GST and catalase activities, specific Et and DCF productions (indicative of intracellular superoxide and peroxide concentrations, respectively) and GSH/GSSG values recorded in stress-exposed and control cultures of *A. nidulans *at 0, 1 and 6 h cultivation times. Click here for file

Additional File 2 List of oxidative stress responsive gene probes. The Excel Table contains basically 3 sets of data: 1. "Gene probes – identification and functional analysis" including "Function categories" and subcategories, "OSU contig name (PipeOnline)" [79], "AN number (Broad Institute)" [80], "Function description" including NCBI Entrez sequence number and data base accession number [84] and "Homology Expect value" [84]. 2. "LOESS normalized M (M') values" for diamide (15 min, 30 min, 1 h, 3 h, 6 h), H_2_O_2 _(15 min, 30 min, 1 h, 3 h, 6 h, 9 h) and menadione (30 min, 1 h, 3 h, 6 h, 9 h) 3. "Responses to stress-inducing agents and physiological parameters" including "More than two-fold transcriptional stress response in the presence of" D (diamide) H (H_2_O_2_) and menadione (M), "Likely responsiveness to changes in the physiological parameter" peroxide, superoxide or GSH/GSSG, "Recovery phase response" in the presence of diamide (D), "Recovery phase response" in the presence of H_2_O_2 _(H) and the indication if a data is "Considered in gene distribution analyses (1502 gene probes)". Click here for file

Additional File 3 Gene probes considered in significant enrichment calculations. Similar to Additional file 2:Supplement2 for the list of oxidative stress responsive gene probes, this Excel Table includes 3 sets of data: 1. "Gene probes – identification and functional analysis" including "Function categories" and subcategories, "OSU contig name (PipeOnline)" [79], "AN number (Broad Institute)" [80], "Function description" including NCBI Entrez sequence number and data base accession number [84] and "Homology Expect value" [84]. 2. "LOESS normalized M (M') values" for diamide (15 min, 30 min, 1 h, 3 h, 6 h), H_2_O_2 _(15 min, 30 min, 1 h, 3 h, 6 h, 9 h) and menadione (30 min, 1 h, 3 h, 6 h, 9 h) 3. "Responses to stress-inducing agents and physiological parameters" including "More than two-fold transcriptional stress response in the presence of" D (diamide) H (H_2_O_2_) and menadione (M), "Likely responsiveness to changes in the physiological parameter" peroxide, superoxide or GSH/GSSG, "Recovery phase response" in the presence of diamide (D) and "Recovery phase response" in the presence of H_2_O_2 _(H). All these data were considered in significant enrichment calculations (Figure 7). Click here for file

Additional File 4 Results of significant enrichment calculations Overrepresentation (highlighted by red) and underrepresentation (highlighted by green) of gene probes with induced, repressed and unchanged expression among diamide, H_2_O_2 _and menadione responsive gene probes (**Part A)**, and among gene probes repressed or induced by changes in the physiological parameters GSH/GSSG, peroxide and superoxide (**Part B**). The following major physiological function categories were considered: I, "Function-unknown"; II, "Signal generation and transduction, DNA transcription, regulation"; III, "Replication, cell division cycle and development"; IV, "RNA splicing and translation, protein maturation"; V, "Defense and stress proteins, degradation of xenobiotics; VI, "Transport, cytoskeleton, cell wall"; VII, "Carbon metabolism"; VIII, "Nitrogen and sulfur metabolism"; IX, "Secondary metabolism"; X, "Oxidoreductases". n stands for the number of gene probes considered in a given function category, and *p *values ≤ 0.05 are indicative of statistical significance. For menadione-induced "Secondary metabolism" gene probes, a *p *value of 0.06 was calculated, which was regarded as statistically significant in this case. Note that one gene probe has only one annotated physiological function in this evaluation scheme. Click here for file

Additional File 5 A selection of genes likely responsive to GSH/GSSG redox imbalance. Gene probes equally up-regulated or down-regulated under diamide, H_2_O_2 _and menadione treatments were regarded as GSH/GSSG responsive. All GSH/GSSG responsive gene probes are presented in Additional file 2:Supplement2 for the list of oxidative stress responsive gene probes and in Additional file 3:Supplement3 for the list of gene probes considered in significant enrichment calculations. Click here for file

Additional File 6 A selection of genes likely responsive to ROS (O_2_^2-^, O_2_^•-^) Gene probes equally up-regulated or down-regulated under H_2_O_2 _and menadione treatments but not responsive to diamide or solely responsive to menadione were regarded as O_2_^2- ^and O_2_^•- ^responsive, respectively. All ROS responsive gene probes are presented in Additional file 2:Supplement2 for the list of oxidative stress responsive gene probes and in Additional file 3:Supplement3 for the list of gene probes considered in significant enrichment calculations. Click here for file
