# Supplementary material for: Comparison of gene expression signatures of diamide, H2O2 and menadione exposed Aspergillus nidulans cultures – linking genome-wide transcriptional changes to cellular physiology
Source: BMC Genomics. 2005 Dec 20;6:182. doi: 10.1186/1471-2164-6-182 (PMC1352360; doi:10.1186/1471-2164-6-182)
Supplement: Additional File 1 — Physiological changes recorded at selected dimide, H2O2 and menadione concentrations and exposure times. DLM values and concentrations selected for DNA microarray experiments are shown for diamide, H2O2 and menadione together with specific SOD, GST and catalase activities, specific Et and DCF productions (indicative of intracellular superoxide and peroxide concentrations, respectively) and GSH/GSSG values recorded in stress-exposed and control cultures of A. nidulans at 0, 1 and 6 h cultivation times. [file 1471-2164-6-182-S1.doc]

**Physiological changes recorded at selected dimide, H_2_O_2_ and menadione concentrations and exposure times ^1^.**

|  | c_selected_  (mM) | DLM  (mM) | Exposure  time (h) | SOD^2^  {U (mg protein)^-1^} | GST  {μkat (kg protein)^-1^} | Catalase  {kat (kg protein)^-1^} | Et^3^  {nmol (g DCM)^-1^} | DCF^3^  {pmol (g DCM)^-1^} | GSH/GSSG |
| --- | --- | --- | --- | --- | --- | --- | --- | --- | --- |
| Control |  |  | 0 | 10 ± 3 | 53 ± 8 | 0.23 ± 0.03 | 37 ± 4 | 800 ± 120 | 78 ± 7 |
|  |  |  | 1 | 12 ± 3 | 50 ± 6 | 0.24 ± 0.02 | 40 ± 4 | 880 ± 180 | 80 ± 8 |
|  |  |  | 6 | 11 ± 2 | 55 ± 6 | 0.26 ± 0.02 | 45 ± 3 | 700 ± 170 | 78 ± 9 |
|  |  |  |  |  |  |  |  |  |  |
| Diamide | 1.8 | > 3.0 | 1 | 17 ± 4 | 70 ± 8^**^ | 0.42 ± 0.05^**^ | 38 ± 2 | 650 ± 120 | 7 ± 2^***^ |
|  |  |  | 6 | 30 ± 5^***^ | 90 ± 10^***^ | 0.9 ± 0.1^***^ | 42 ± 4 | 700 ± 130 | 37 ± 5^***^ |
|  |  |  |  |  |  |  |  |  |  |
| H_2_O_2_ | 75 | 500 | 1 | 15 ± 3 | 60 ± 6 | 0.35 ± 0.04^**^ | 33 ± 4 | 1600 ± 300^***^ | 11 ± 2^***^ |
|  |  |  | 6 | 19 ± 3^*^ | 75 ± 7^**^ | 0.41 ± 0.04^**^ | 35 ± 4 | 700 ± 100 | 85 ± 7 |
|  |  |  |  |  |  |  |  |  |  |
| Menadione | 0.8 | 1.4 | 1 | 45 ± 8^***^ | 85 ± 6^***^ | 0.50 ± 0.06^***^ | 47 ± 4^*^ | 2300 ± 170^***^ | 73 ± 12 |
|  |  |  | 6 | 80 ± 8^***^ | 100 ± 6^***^ | 0.8 ± 0.1^***^ | 99 ± 7^***^ | 3660 ± 660^***^ | 44±10^***^ |

^1^ - Specific enzyme activity and production values and GSH/GSSG ratios are expressed as mean ± S.D. calculated from 4 independent experiments.

^2^ - One U of SOD activity was defined as the amount of enzyme that inhibited the Nitro Blue Tetrazolium oxidation rate of the control by 50 %.

^3^ - Specific Et and DCF productions are indicative of intracellular superoxide and peroxide concentrations, respectively.

^*^ - *P*< 5 %; ^**^ - *P*< 1 %; ^***^ - *P* < 0.1 %.
