# Supplementary material for: Comparison of gene expression signatures of diamide, H2O2 and menadione exposed Aspergillus nidulans cultures – linking genome-wide transcriptional changes to cellular physiology
Source: BMC Genomics. 2005 Dec 20;6:182. doi: 10.1186/1471-2164-6-182 (PMC1352360; doi:10.1186/1471-2164-6-182)
Supplement: Additional File 5 — A selection of genes likely responsive to GSH/GSSG redox imbalance. Gene probes equally up-regulated or down-regulated under diamide, H2O2 and menadione treatments were regarded as GSH/GSSG responsive. All GSH/GSSG responsive gene probes are presented in Additional file 2:Supplement2 for the list of oxidative stress responsive gene probes and in Additional file 3:Supplement3 for the list of gene probes considered in significant enrichment calculations. [file 1471-2164-6-182-S5.doc]

**A selection of genes likely responsive to GSH/GSSG redox imbalance^1^**

| **Physiological functions Genes responsive to oxidative stress^2,3^**  **Gene [organism harboring closest homologue], OSU contig collection ID,**  **Broad Institute ORF ID (Induction or Repression)** | |
| --- | --- |
| Signal generation and transduction, DNA transcription, regulation | - putative PBS2 like MAPK kinase gene homologue [*Neurospora crassa*], contig2000Sep131300_3655, AN0931.2 (I)  - *atfA* bZip domain-containing transcriptional factor [*Aspergillus nidulans*], contig2000Sep131300_3103, AN2911.2 (I)  - *meaB* nitrogen metabolic repressor of *Aspergillus nidulans*, AN4900.2 (I)^4,5^  - putative ubiquitin carboxyl-terminal hydrolase [*Schizosaccharomyces pombe*], contig2000Sep131300_3068, AN6354.2 (I)  - *UBI4* polyubiquitin [*Saccharomyces cerevisiae*], contig2000Sep131300_1727, AN2000.2 (I)  - *ubr1* N-end-recognizing protein (ubiquitin-protein ligase E3 component) [*Schizosaccharomyces pombe*], contig2000Sep131300_694, AN3923.2 (I)  - *PSK2* (PAS domain-containing serine/threonine kinase) homologue [*Saccharomyces cerevisiae*], contig2000Sep131300_4391, AN4536.2 (R)  - *hhk7* putative histidine kinase [*Cochliobolus heterostrophus*], contig2000Sep131300_802, AN2363.2 (R)  - *prnA* positive regulator of Pro degradation of *Aspergillus nidulans*, AN1729.2 (R)^5^ |
| Replication, cell division cycle and development | - *uvsF* - replication factor C like protein of *Aspergillus nidulans*, AN6303.2 (I)^4,5^  - *PPS1* protein phosphatase S phase [*Saccharomyces cerevisiae*], contig2000Sep131300_1836, AN0129.2 (I)  - *pphA* protein phosphatase 2a [*Aspergillus nidulans*], contig2000Sep131300_2903, no AN ORF found (I)  - *HOS4* subunit of the Set3 complex (meiotic-specific repressor) [*Saccharomyces cerevisiae*], contig2000Sep131300_4593, AN4072.2 (I)  - histone H2B [*Aspergillus nidulans*], contig2000Sep131300_1655, AN3469.2 (R)  - putative helicase [*Schizosaccharomyces pombe*], contig2000Sep131300_97, AN3444.2 (R)  - *rad26* DNA repair protein [*Neurospora crassa*], contig2000Sep131300_2413, AN3811.2 (R) |
| RNA splicing and translation, protein maturation | - putative RNA-binding protein [*Schizosaccharomyces pombe*], contig2000Sep131300_2799, AN7145.2 (I)  - 40s ribosomal protein S4-2 [*Schizosaccharomyces pombe*], contig2000Sep131300_2159, AN4794.2 (I)  - putative translation initiation factor eif-2b beta subunit gene homologue [*Schizosaccharomyces pombe*], contig2000Sep131300_3764, AN1344.2 (I)  - elongation factor 3 [*Aspergillus fumigatus*], contig2000Sep131300_867, AN6700.2 (I)  - putative mRNA stability protein [*Schizosaccharomyces pombe*], contig2000Sep131300_1771, AN6136.2 (R)  - glycyl tRNA synthetase [*Schizosaccharomyces pombe*], contig2000Sep131300_4237, AN8835.2 (R)  -*crp2* (40S ribosomal protein S14) [*Neurospora crassa*], contig2000Sep131300_1471, AN5960.2 (R)  - 40S ribosomal protein S15 (S12) [*Podospora anserina*], contig2000Sep131300_1511, AN5997.2 (R)  - *crp3*(40s ribosomal protein S17) [*Neurospora crassa*], contig2000Sep131300_1350, AN5979.2 (R)  - 40S ribosomal protein S22 (S15A) (YS24) [*Neurospora crassa*], contig2000Sep131300_1807, AN0907.2 (R)  - ribosomal protein Srp1 [*Sclerotinia sclerotiorum*], contig2000Sep131300_25 and contig2000Sep131300_1605, AN4802.2 (R)  - 60S ribosomal protein L25 [*Puccinia graminis*], contig2000Sep131300_3698, AN8856.2 (R)  - *RPL33B* ribosomal protein L37 of the large (60S) ribosomal subunit [*Saccharomyces cerevisiae*], contig2000Sep131300_1203, AN2980.2 (R)  - *GPI8* ER membrane glycoprotein subunit of the glycosylphosphatidylinositol transamidase complex [*Saccharomyces cerevisiae*], contig2000Sep131300_2304, AN0871.2 (R) |
| Defense and stress proteins, degradation of xenobiotics | - glutathione peroxidase [*Blumeria graminis*], contig2000Sep131300_103, AN2846.2 (I)^4^  - heat shock protein CLPA [*Paracoccidioides brasiliensis*], contig2000Sep131300_1696, AN0858.2 (I)  - heat shock protein 80 [*Neurospora crassa*], contig2000Sep131300_3947, AN8269.2 (I)  - α,α-trehalose-phosphate synthase [*Schizosaccharomyces pombe*], contig2000Sep131300_1551, AN4262.2 (I)  - trehalose synthase (clock controlled gene-9) [*Neurospora crassa*], contig2000Sep131300_557, AN5021.2 (I)  - *treB* neutral trehalase of *Aspergillus nidulans*, AN5635.2 (R)^5^  - matrix AAA protease MAP-1 [*Neurospora crassa*], contig2000Sep131300_3516, AN4557.2 (R)  - putative chlorohydrolase/deaminase [*Schizosaccharomyces pombe*], contig2000Sep131300_2397, AN3194.2, (R)  - probable hydroxyquinol-1, 2-dioxygenase [*Neurospora crassa*], contig2000Sep131300_4539, AN9363.2 (R) |
| Transport, cytoskeleton, cell wall | - clathrin light chain [*Schizosaccharomyces pombe*], contig2000Sep131300_940, AN2050.2 (I)  - synaptobrevin [*Aspergillus parasiticus*], contig2000Sep131300_1495, AN8769.2 (I)  - *VPS21* Rab5-like GTPase [*Saccharomyces cerevisiae*], contig2000Sep131300_3122, AN4915.2 (I)  - *OSH1* Oxysterol-binding protein homolog 1 [*Saccharomyces cerevisiae*], contig2000Sep131300_2880, AN9063.2 (I)  - *itr1* myo-inositol transport protein [*Neurospora crassa*], contig2000Sep131300_2485, AN3210.2 (I)  - P-type Na^+^-ATPase [*Fusarium oxysporum* f. sp*. lycopersici*], contig2000Sep131300_4499, AN6642.2 (I)  - P-type ATPase [*Neurospora crassa*], contig2000Sep131300_3305, AN7664.2 (I)  - vacuolar ATP Synthase 98 kD subunit [*Neurospora crassa*], contig2000Sep131300_2830, AN5606.2 (I)  - *abcF2* non-transporter ABC protein [*Dictyostelium discoideum*], contig2000Sep131300_1467, AN2210.2 (I)  - putative nucleoporin [*Schizosaccharomyces pombe*], contig2000Sep131300_2982, AN2086.2 (R)  - kinesin [*Cochliobolus heterostrophus*], contig2000Sep131300_3093, AN7547.2 (R) |
| Carbon metabolism | - 6-phosphofructo-2-kinase [*Neurospora crassa*], contig2000Sep131300_523, AN5144.2 (I)  - putative 2-hydroxyacid dehydrogenase [*Schizosaccharomyces pombe*], contig2000Sep131300_2761, AN0775.2 (I)  - putative short-chain alchohol dehydrogenase [*Aspergillus oryzae*], contig2000Sep131300_1560, AN7817.2 (I)  - fructose biphosphate aldolase [*Paracoccidioides brasiliensis*], contig2000Sep131300_628, AN2875.2 (R)  - *adh1* alcohol dehydrogenase I [*Aspergillus nidulans*], contig2000Sep131300_1664 and contig2000Sep131300_1677, AN8979.2 (R)  - phosphatidate cytidylyltransferase [*Schizosaccharomyces pombe*], contig2000Sep131300_3859, AN5166.2 (R) |
| Nitrogen and sulphur metabolism | - glutamic acid decarboxylase [*Aspergillus oryzae*], contig2000Sep131300_1993, AN5447.2 (I)  - cobalamin-independent methionine synthase [*Aspergillus nidulans*], contig2000Sep131300_3136, AN4443.2 (R)  - aldehyde dehydrogenase family 6, subfamily A1 [*Mus musculus*], contig2000Sep131300_144, AN3591.2 (R)  - S-adenosylmethionine synthetase [*Neurospora crassa*], contig2000Sep131300_929, AN1222.2 (R)  - kynurenine 3-monooxygenase [*Bombyx mori*], contig2000Sep131300_385, AN5200.2 (R)  - inosine 5'-monophosphate dehydrogenase [*Pneumocystis carinii*], contig2000Sep131300_4331, no AN ORF found (R)  - *hxB* molybdenum cofactor sulfurase protein of *Aspergillus nidulans*, AN1637.2 (R)^5^ |
| Secondary metabolism | - *stcI* putative sterigmatocystin biosynthesis lipase/esterase of *Aspergillus nidulans*, AN7816.2 (I)^5^  - *stcS* putative demethylsterigmatocystin synthase [*Aspergillus nidulans*], contig2000Sep131300_844, AN7808.2 (I)  - trichothecene C-15 hydroxylase [*Gibberella zeae*], contig2000Sep131300_3125, AN7881.2 (I)  - polyketide synthase [*Botryotinia fuckeliana*], contig2000Sep131300_4090, AN2403.2 (I)  - putative 4-coumarate-CoA ligase, putative [*Arabidopsis thaliana*], contig2000Sep131300_4200, AN5990.2 (R)^4^ |
| Oxidoreductases - respiration | - NADH-ubiquinone oxidoreductase 29.9 KD subunit [*Neurospora crassa*], contig2000Sep131300_3583, AN5971.2 (I)  - putative flavoprotein subunit [*Schizosaccharomyces pombe*], contig2000Sep131300_1281 and contig2000Sep131300_3424, AN1543.2 (R) |

^1^ - All GSH/GSSG responsive gene probes are presented in Additional file 1:Supplement1 for the list of oxidative stress responsive gene probes and in Additional file 2:Supplement2 for the list of gene probes considered in significant enrichment calculations

^2^ - In the case of function-not-yet-identified genes, closest homologues were found *via* translated ORF query *versus* protein in NCBI BLAST (blastp) [84].

^3^ - For diamide and H_2_O_2_, only early (0.25-1.0 h exposure times) transcriptional changes were analysed and recovery phase data (3-9 h) were disregarded. More information including size and time-dependence of transcriptional changes is available at NCBI GEO [13] on Platforms GPL1752 and GPL1756, and also shown in Additional file 1:Supplement1 for the list of oxidative stress responsive gene probes.

^4^ - A more than two-fold transcriptional change is observable at 1 h H_2_O_2_ treatment but no data are available at 0.25 and 0.5 h H_2_O_2_ treatments.

^5^ - Sequence printed onto the chips was PCR-amplified from a custom-made cDNA plasmid library [10] with 2 gene specific primers.
