# Supplementary material for: Comparison of gene expression signatures of diamide, H2O2 and menadione exposed Aspergillus nidulans cultures – linking genome-wide transcriptional changes to cellular physiology
Source: BMC Genomics. 2005 Dec 20;6:182. doi: 10.1186/1471-2164-6-182 (PMC1352360; doi:10.1186/1471-2164-6-182)
Supplement: Additional File 6 — A selection of genes likely responsive to ROS (O22-, O2•-) Gene probes equally up-regulated or down-regulated under H2O2 and menadione treatments but not responsive to diamide or solely responsive to menadione were regarded as O22- and O2•- responsive, respectively. All ROS responsive gene probes are presented in Additional file 2:Supplement2 for the list of oxidative stress responsive gene probes and in Additional file 3:Supplement3 for the list of gene probes considered in significant enrichment calculations. [file 1471-2164-6-182-S6.doc]

**A selection of genes likely responsive to ROS (O_2_^2-^, O_2_^•-^)^1^**

| **Physiological functions Genes responsive to oxidative stress^2,3^**  **Gene [organism harboring closest homologue], OSU contig collection ID,**  **Broad Institute ORF ID (ROS; Induction or Repression)** | |
| --- | --- |
| Signal generation and transduction, DNA transcription, regulation | - putative histidine kinase *hhk4* [*Cochliobolus heterostrophus*], contig2000Sep131300_2835, AN2581.2 (O_2_^2-^, I)  - *cpcA* cross-pathway control protein [*Aspergillus nidulans*], contig2000Sep131300_1641, AN3675.2 (O_2_^2-^, I)  - *PbLON* lon proteinase gene [*Paracoccidioides brasiliensis*], contig2000Sep131300_2597, AN6193.2 (O_2_^•-^, I)  - *jlbA* JUN-like bZIP transcription factor [*Aspergillus nidulans*], contig2000Sep131300_3712, AN1812.2 (O_2_^2-^, R)  - *cppk1* protein kinase 1 [*Cryphonectria parasitica*], contig2000Sep131300_3958, AN5973.2 (O_2_^•-^, R)  - related to transcription adaptor *ADA2* [*Neurospora crassa*], contig2000Sep131300_4436, AN5974.2 (O_2_^•-^, R) |
| Replication, cell division cycle and development | - related to ser/thr protein kinase *IME2* [*Neurospora crassa*], contig2000Sep131300_3907, AN6243.2 (O_2_^2-^, I)  - β-transducin-like GTP binding protein HET-D2Y [*Podospora anserina*], contig2000Sep131300_3183, AN2021.2 (O_2_^2-^, I)  - exonuclease II [*Schizosaccharomyces pombe*], contig2000Sep131300_3529, AN8185.2 (O_2_^•-^; I)  - *aspC* septin [*Aspergillus nidulans*], contig2000Sep131300_4182, AN8182.2 (O_2_^•-^; R)  - *cdc5* [*Lentinula edodes*], contig2000Sep131300_3154, AN7495.2 (O_2_^•-^; R)  - *spoC1-C1C* conidium-specific gene [*Aspergillus nidulans*], contig2000Sep131300_4354, AN5085.2 (O_2_^•-^; R) |
| RNA splicing and translation, protein maturation | - putative Upf1p-interacting protein gene *NMD3* [*Saccharomyces cerevisiae*], contig2000Sep131300_4240, AN1711.2 (O_2_^2-^, I)  - phenylalanyl-tRNA synthetase  β-chain [*Candida albicans*], contig2000Sep131300_2534, AN4086.2 (O_2_^2-^, I)  - *cpcB* Gβ like protein [*Aspergillus nidulans*], contig2000Sep131300_237, AN4163.2 (O_2_^•-^; R) |
| Defense and stress proteins, degradation of xenobiotics | - *sodA* Cu,Zn-superoxide dismutase [*Aspergillus nidulans],* contig2000Sep131300_575, AN0241.2 (O_2_^•-^; I)  - dienelactone hydrolase [*Chaetomium globosum*], contig2000Sep131300_4300, AN3712.2 (O_2_^•-^; I)  - *catA* catalase A (spore-specific catalase) [*Aspergillus nidulans*], contig2000Sep131300_996, AN8637.2 (O_2_^•-^; R) |
| Transport, cytoskeleton, cell wall | - *HXT1* hexose transporter [*Saccharomyces cerevisiae*], contig2000Sep131300_731, AN9295.2 (O_2_^2-^, I)  - yeast *GTR2* homologue, novel small GTPase subfamily protein [*Schizosaccharomyces pombe*], contig2000Sep131300_3536, AN0953.2 (O_2_^•-^; I)  - *vpsB* Sec1-like protein [*Aspergillus nidulans*], contig2000Sep131300_2794, AN6531.2 (O_2_^2-^, R)  - *ntf2* nuclear transport factor 2 [*Aspergillus nidulans*], contig2000Sep131300_950, AN4942.2 (O_2_^2-^, R)  - MFS transporter [Beauveria bassiana], contig2000Sep131300_163, AN2831.2 (O_2_^2-^, R)  - *benA* β-tubulin of *Aspergillus nidulans*^3^, AN1182.2 (O_2_^•-^; R)  - *hxtA* high-affinity hexose transporter [*Aspergillus nidulans*], contig2000Sep131300_4232, AN6923.2 (O_2_^•-^; R)  - ABC transporter protein [*Aspergillus nidulans*], contig2000Sep131300_2666, AN7581.2 (O_2_^•-^; R)  - putative ABC transporter [*Mycosphaerella graminicola*], contig2000Sep131300_2424, AN3952.2 (O_2_^•-^; R)  - putative membrane transporter [*Schizosaccharomyces pombe*], contig2000Sep131300_2681, AN9135.2 (O_2_^•-^; R)  - putative endosomal Vps protein complex subunit [*Schizosaccharomyces pombe*], contig2000Sep131300_1373, AN6920.2 (O_2_^•-^; R)  - *ran/spi1* GTP-binding protein [*Schizosaccharomyces pombe*], contig2000Sep131300_1901, AN0084.2 (O_2_^•-^; R)  - mannosidase, required for cell wall biosynthesis [*Schizosaccharomyces pombe*], contig2000Sep131300_4532, AN0393.2 (O_2_^•-^; R) |
| Carbon metabolism | - geranylgeranyl pyrophosphate synthetase (GGPP synthetase) [*Neurospora crassa*], contig2000Sep131300_3434, AN6810.2 (O_2_^2-^, I)  - acetyl-CoA-acyltransferase [*Mycosphaerella graminicola*], contig2000Sep131300_3747, AN4179.2 (O_2_^•-^; I)  - STE1 esterase gene [*Metarhizium anisopliae* var. *anisopliae*], contig2000Sep131300_1264, AN8528.2 (O_2_^•-^; I)  - α-glucosidase [*Aspergillus oryzae*], contig2000Sep131300_3700, AN4843.2 (O_2_^2-^, R)  - *cbhA* 1,4-β-D-glucan-cellobiohydrolyase [*Aspergillus nidulans*], contig2000Sep131300_1738, no AN ORF found (O_2_^2-^, R)  - *pdcA* pyruvate decarboxylase [*Aspergillus nidulans*], contig2000Sep131300_1324, AN4888.2 (O_2_^2-^, R)- lanosterol synthase related protein [*Neurospora crassa*], contig2000Sep131300_3318, AN8260.2 (O_2_^•-^; R)  - aldehyde dehydrogenase, mitochondrial precursor (ALDH class 2) [*Leishmania tarentolae*], contig2000Sep131300_2192, AN9198.2 (O_2_^•-^; R) |
| Nitrogen and sulphur metabolism | - *spdA* spermidine synthase [*Aspergillus nidulans*], contig2000Sep131300_3359, AN0687.2 (O_2_^2-^, I)  - *shm2* serine hydroxymethyltransferase [*Schizosaccharomyces pombe*], contig2000Sep131300_549, AN5835.2 (O_2_^2-^, I)  - *mccB* non-biotin containing subunit of 3-methylcrotonyl CoA carboxylase [*Aspergillus nidulans*], contig2000Sep131300_4571, AN4687.2 (O_2_^•-^; I)  - pepstatin-insensitive acid protease [*Talaromyces emersonii*], contig2000Sep131300_2414, AN3377.2 (O_2_^•-^; R) |
| Secondary metabolism | - cytochrome P450 monooxygenase [*Gibberella fujikuroi*], contig2000Sep131300_3216, AN9253.2 (O_2_^2-^, I)  - monooxigenase [*Aspergillus parasiticus*], contig2000Sep131300_2509, AN5421.2 (O_2_^•-^; I) |
| Oxidoreductases - respiration | - complex I intermediate associated protein CIA30 homologue [*Aspergillus nidulans*], contig2000Sep131300_4114, AN4740.2 (O_2_^2-^, I) |

^1^ - All ROS responsive gene probes are presented in Additional file 1:Supplement1 for the list of oxidative stress responsive gene probes and in Additional file 2:Supplement2 for the list of gene probes considered in significant enrichment calculations

^2^ - In the case of function-not-yet-identified genes, closest homologues were found *via* translated ORF query *versus* protein in NCBI BLAST (blastp) [84].

^3^ - For diamide and H_2_O_2_, only early (0.25-1.0 h exposure times) transcriptional changes were analysed and recovery phase data (3-9 h) were disregarded. More information including size and time-dependence of transcriptional changes is available at NCBI GEO [13] on Platforms GPL1752 and GPL1756, and also shown in Additional file 1:Supplement1 for the list of oxidative stress responsive gene probes.

^4^ - Sequence printed onto the chips was PCR-amplified from a custom-made cDNA plasmid library [10] with 2 gene specific primers.
